# Multi-omics of extracellular vesicles: An integrative representation of functional mediators and perspectives on lung disease study

**DOI:** 10.3389/fbinf.2023.1117271

**Published:** 2023-02-09

**Authors:** Yuexing Liu, Yixue Li, Tao Zeng

**Affiliations:** Guangzhou Laboratory, Guangzhou, China

**Keywords:** extracellular vesicles, multi-omics, integration, lung disease, integrative bioinformatics

## Abstract

Extracellular vesicles are secreted by almost all cell types. EVs include a broader component known as exosomes that participate in cell–cell and tissue–tissue communication *via* carrying diverse biological signals from one cell type or tissue to another. EVs play roles as communication messengers of the intercellular network to mediate different physiological activities or pathological changes. In particular, most EVs are natural carriers of functional cargo such as DNA, RNA, and proteins, and thus they are relevant to advancing personalized targeted therapies in clinical practice. For the application of EVs, novel bioinformatic models and methods based on high-throughput technologies and multi-omics data are required to provide a deeper understanding of their biological and biomedical characteristics. These include qualitative and quantitative representation for identifying cargo markers, local cellular communication inference for tracing the origin and production of EVs, and distant organ communication reconstruction for targeting the influential microenvironment and transferable activators. Thus, this perspective paper introduces EVs in the context of multi-omics and provides an integrative bioinformatic viewpoint of the state of current research on EVs and their applications.

## Introduction

Extracellular vesicles (EVs) are membranous nanoparticles secreted by almost all cell types. With some similarity to circulating DNA (ctDNA) and circulating cancer cells (CTCs), EVs that contain a molecular cargo can now be extracted from body fluids using sensitive devices and platforms (Tellez-Gabriel et al., 2020). As a broad representative class of EVs (with diameters ranging from 30 to 150 nm) delivered to the extracellular space, exosomes participate in intercellular and intra-organ communication (Ghafourian et al., 2022). As such, they are capable of carrying diverse biological signals from one cell type or tissue to another. The exosomal cargo includes proteins, lipids, miRNAs, and other ncRNAs (Isaac et al., 2021). Currently, there are many comprehensive bioinformatic databases available for understanding EVs, for example, the EVAtlas containing the most comprehensive ncRNA expression in EVs (Liu et al., 2022), and exoRBase identifying novel EVs and long RNA signatures from human biofluids (Lai et al., 2022).

On the one hand, the most recent studies and developments indicate the possibility and feasibility of adopting bioinformatic methods to study EVs. Many existing tools from bioinformatics have been applied to identify key functional cargo in EV datasets. There are web-based resources for elucidating molecular mechanisms and pathophysiology of EVs isolated from different disease conditions, including ExoCarta, EVpedia, and Vesiclepedia (Keerthikumar et al., 2017). The miRanda, PITA, and RNAhybrid programs can be used to identify differentially expressed microRNAs derived from exosomes and their potential target genes (Zeng et al., 2022). There are many bioinformatics tools available for evaluating various parameters relevant to EVs, and these analyses can help to identify the functional ability of EVs by analyzing host-pathogen interactions, toxicity, omics, and pathogenesis (Saravanakumar et al., 2022). On the other hand, new omics technology and multi-omics methods are required to translate basic biological information of EVs into urgently needed clinical applications. Increasing numbers of studies have revealed the contributions of EVs to carcinogenesis, metastasis, and the immunological response (Papadakos et al., 2022). EVs are involved in many biological processes (Zhang et al., 2022), and they act as communication messengers within the intercellular network, mediating different physiological activities or pathological changes (Zhao et al., 2022). In particular, EVs are natural carriers of functional proteins, RNA, and DNA, and thus they could efficiently deliver key molecules and drugs as cost-efficient therapeutic tools (Pang et al., 2020). Indeed, EVs and related developments in various omics analyses should be helpful for the early diagnosis of tumors (Gongye et al., 2022) and the development of personalized targeted therapies in clinical practice (Papadakos et al., 2022).

**FIGURE 1:**
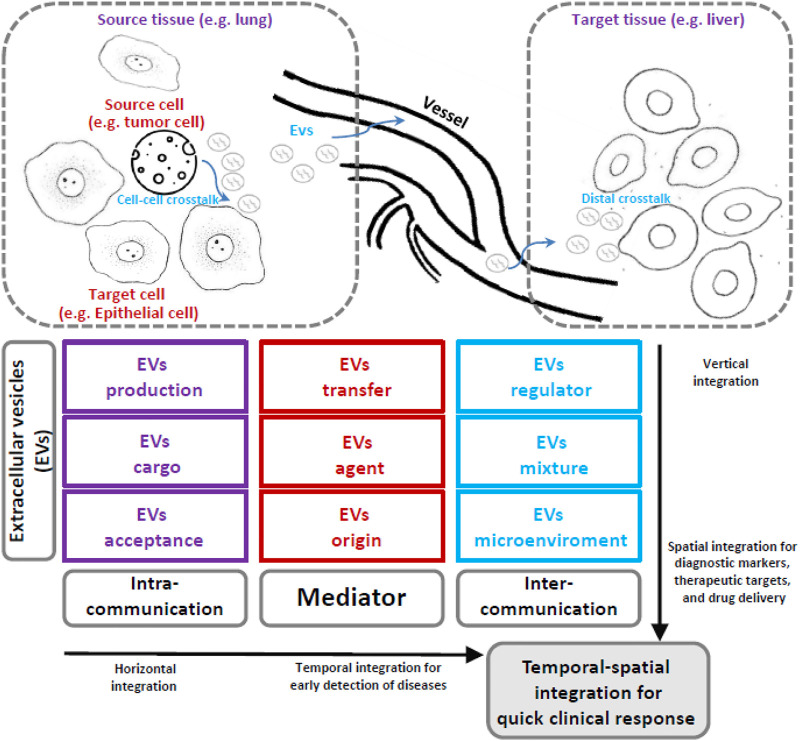
The integrative bioinformatic research ideas and the workflow of extracellular vesicles. EVs have many biological functions related to their specific roles in physiological and pathological processes. On the one hand, EVs can participate in cell–cell communication, e.g., the EV production, cargo packaging, and signal transduction among cells together can implement cell–cell communication that could be spatially characterized by vertical integration and provide a series of novel biomarkers. On the other hand, the mediator role of EVs is a key component of tissue–tissue communication. For example, the EV secretion, transfer, and regulation from the source tissue to the distant target tissue consist of a many-to-many communication network that could be temporally traced by horizontal integration and thereby provide early warning biomarkers. Similarly, many EVs with a specific cargo (satisfactory EV acceptance) may be transferred by specific agents (e.g., exosomes with a particular origin) that could further create a microenvironment in target tissues comprising a mixture of different EVs. Given these characteristics of EVs, the integrative bioinformatic approach has great possibility and feasibility to reveal the temporal-spatial patterns of EVs and associated biomarkers, a characteristic that is especially suitable for supporting a rapid response in clinical applications, for example, in treating major respiratory infectious diseases.

The next direction of EVs should be a deeper understanding of their biological and biomedical characteristics *via* multi-omics analysis (Poh et al., 2023). This will require the corresponding support from bioinformatic modeling and methods using an integrative viewpoint (Figure 1). i) EVs work as mediators, and their cargo includes many kinds of biological molecules; therefore, multi-omics data and representation should be keys to qualitatively and quantitatively describing their functional roles. ii) EVs are produced from biological information senders (i.e., source cells or tissues), and their cross-talk within local cellular communication networks could be clues by which to trace their origin and production. iii) EVs also transfer and influence various biological information receivers (target cells or tissues), and their cross-talk within remote organ communication networks should be valuable for activating the remote target microenvironment.

Thus, this paper aims to introduce EVs in a multi-omics context and to provide a perspective on their current research status and diverse clinical applications (e.g., prevention, diagnosis, and prognosis for lung diseases) from the viewpoint of integrative bioinformatics.

## Functional communication at the cell level

The nanosized EVs can be secreted from almost all existing cell types (Kucuk et al., 2021; Wang et al., 2022), and they participate in cell–cell communication (Feng et al., 2021; van Niel et al., 2022). In a study of the EVs spontaneously flowing between non-stem cancer cells and cancer stem cells (CSC), tumors were shown to adapt and thrive depending on a cellular collaboration network mediated by EVs secreted from the CSC (Ruivo et al., 2022). Although a recent work suggested that EV-borne miRNAs would be minor effectors of the stability of the recipient cells’ transcriptome (Albanese et al., 2021), the EV-capsuled factors can regulate cancer hallmarks during tumorigenesis locally and also enter the circulation to distant organs *via* the construction of a pre-metastatic niche and further metastasis (Chang and Pauklin, 2021; Zhang et al., 2022).

Many kinds of molecular cargo in EVs should have respective roles in the above biological or biomedical procedures and thus can be comprehensively characterized and represented by multi-omics datasets and bioinformatic methods. A series of bioinformatic approaches have been applied to identify biomarkers in EVs with possible immunological clinical value (Wang and Yu, 2020). These include differential expression analysis for marker genes, prognostic analysis by SurvExpress, transcription factor networks predicted by NetworkAnalysis, a distinct association of immune cells between cells, and EVs characterized by single-sample gene set enrichment analysis. Biomarker candidates for disease can also be detected by combining miRNA-mediated competitive regulation and differential expression analysis, depending on the EVs’ specific ceRNA network inferred by integrating the transcriptome data and lncRNA regulatory association data (Fang et al., 2022). In addition, cell–cell communication has been understood by combined proteomics, lipidomics, and metabolomics of EVs from infected macrophages; this can help design therapeutic agents/targets for fighting deadly mycosis (Zhang et al., 2021).

Therefore, integrative bioinformatic models and methods (Yu and Zeng, 2018; Zhang et al., 2022; Tang et al., 2022; Yu et al., 2022) should be suitable for inferring the integrative representation of EVs at the molecular and cellular levels, and this in turn can aid in understanding the intracellular journeys of EVs. As it is not limited to the typical integration of genetic information for examining the potential biological pathways in a cell, the new integrative bioinformatic analysis of EVs can bridge the underlying regulatory signal flow among cells by combining with other cutting-edge biotechnology methods such as single-cell omics using a form of vertical integration. Such integrative omics data analysis based on high-throughput technology should provide new data resources to artificial intelligence for bioscience and further advance the development of novel BT&IT approaches for EVs (Zeng et al., 2021) that can support a unified clinical research chain, including the identification of diagnostic markers, recognition of therapeutic targets, and designs for drug delivery.

## Functional communication at the tissue level

Many EVs have a membrane-enclosed structure, and they carry diverse biological molecules, which can realize the intercellular transfer of functional molecules (e.g., cross-talk between tumors and normal neighboring or remote tissues). The functional ncRNAs and proteins enriched in EVs secreted from tumor tissues could serve as biomarkers for diverse complex diseases (Dong et al., 2020) and possibly act as suitable vectors for drug delivery specific to tumors rather than normal tissues (Liu et al., 2021). For example, the proteins in EVs based on comprehensive EV proteomics are expected to be more enriched in tissue-specific EVs, a factor that is especially helpful for monitoring pre-disease or disease states when patient blood is collected (Muraoka et al., 2022). The content of EVs in placental tissue can also be diagnostic for many metabolic diseases (Szabo, 2021; Jafari et al., 2022; Muraoka et al., 2022) such as gestational hypertension, gestational diabetes mellitus, and preterm birth during pregnancy (Apostolopoulou et al., 2021; Ghafourian et al., 2022; Pavani et al., 2022). In particular, microbiota-released EVs have been shown to play mediator roles in microbiota-host communication and inter-bacterial and inter-kingdom signaling through intercellular signaling mechanisms (Sultan et al., 2021).

Using present multiparametric extraction protocols, multiple omics materials (e.g., DNA, RNA, and protein) can be simultaneously extracted from the same limited starting tissue material (Shaba et al., 2022), thereby guaranteeing the sensitivity and specificity of EVs-based bioinformatic research for disease detection and monitoring (Roy et al., 2021) and further enhancing the diagnostic value of EVs (Chisholm et al., 2022). The multidimensional cargo of EVs should reflect the underlying pathophysiological process. Based on this, the implementation of multi-omics can be used to study the molecular complexity of highly purified EVs and recognize EVs’ specific functions and potential as biomarkers (Dhondt et al., 2020). Notably, a multi-omics study has shown its merit for identifying novel EV-associated biomarkers, for example, Alzheimer’s disease signatures detected by the integrated analysis of 1,000 proteins, 594 lipids, and 105 miRNAs from EVs derived from microglia tissue (Cohn et al., 2021).

Thus, multi-omics analysis based on integrative bioinformatics can strongly support analysis of the joint or multi-view representation of EVs and can aid in understanding the intercellular journeys of EVs. This information could be integrated temporally. The relationship mediated by EVs between information senders and receivers (i.e., from source tissue to target tissue) is generally many-to-many. In particular, the EVs from tumor tissues would carry specific signal molecules that tend to communicate with certain distant tissues/cells (Melo et al., 2015; Becker et al., 2016). Thus, the integrative bioinformatic analysis of EVs indeed can help reconstruct the cargo-specific communication networks among the source and target tissues in addition to cell–cell communication networks. Such analyses can provide new methods for the early detection and treatment of complex diseases of the source tissue to prevent uncontrolled disease deterioration in target tissues.

## Perspectives on lung disease study

With the development and application of EVs, these biological mediators have shown strong potential for battling various lung diseases. First, EVs can help to construct a life cycle scheme to fight against acute respiratory diseases. In plasma, SARS-CoV-2 RNA-positive EVs may provide an alternative diagnostic approach for patients without SARS-CoV-2 RNA detectable in the respiratory tract (Lam et al., 2021; Ning et al., 2021). Second, EVs can help identify convenient health control measures for managing chronic respiratory diseases. Since EVs and their cargo have the potential to modulate common pathological processes (e.g., inflammation, apoptosis, and fibrosis) in different chronic diseases, they could provide new prognostic signatures and therapeutic targets for patients with chronic obstructive pulmonary disease (COPD) (Reid et al., 2021). Third, EVs can be used to implement personalized prognosis and treatment for malignant respiratory diseases. Taking liver metastasis of lung cancer as an example, dysregulated miR-122-5p in non-small-cell lung cancer (NSCLC) cells can promote lung cancer progression by creating a pre-metastatic microenvironment in liver cells for hepatic metastasis (Li et al., 2021).

Based on the above brief perspective concerning the importance of EVs in lung diseases, bioinformatics and multi-omics studies have been used in applications in investigating the biological roles and clinical values of different kinds of EV cargo in lung disease diagnosis and prognosis. Using plasma samples retrieved and analyzed from lung cancer patients, differentially expressed microRNAs were screened by bioinformatic analysis, and their target gene sets were identified by the miRanda, PITA, and RNAhybrid programs and evaluated by function and KEGG pathway enrichment analyses. The results finally determined a promising plasma EVs-derived miRNA target that had an impact on radiotherapy outcomes of NSCLC patients through the Ras signaling pathway (Zeng et al., 2022). Similarly, based on serum-derived Piwi-interacting RNA (piRNA) of EVs from healthy and diseased individuals, a candidate signature piRNA has been screened by differential expression analysis and validated by quantitative real-time PCR, with an assessment by the area under the curve value and associated analysis as to age and the TNM stage of patients. This would be an effective and promising biomarker for the early diagnosis of NSCLC (Li et al., 2022). Meanwhile, an EV protein, fibronectin, can be detected by an efficient bioinformatic analysis and validated by *in silico* immunohistochemical and parallel reaction monitoring; this method was assessed with satisfactory classification accuracy in an independent NSCLC cohort (An et al., 2019). In addition, combined with exosomes purified from lung cancer cells, untargeted metabolic profiling, and metabolic pathways analysis, exosome-based metabolism has demonstrated biomarker identification ability in human biofluids (Choi et al., 2022). In particular, based on a transcriptome and proteome atlas of tumor-derived exosomes, integrative bioinformatic analysis has detected diverse exosomal-enriched RNAs and proteins and their tumorigenesis-associated regulatory mechanisms in mediating lung cancer development (Luo et al., 2021).

Collectively, EVs have paved the way for the early detection and treatment of complex diseases such as cancer (Crosby et al., 2022; Fitzgerald et al., 2022). Along with the development of technologies working with small messengers such as EVs, the cellular and molecular mechanisms governing many observed functions of EVs can be resolved, meaning that EVs would be fully identified as disease biomarkers, therapeutic agents, and drug delivery vehicles for human health and complex diseases (van Niel et al., 2022). Meanwhile, data-intensive scientific research (Zheng et al., 2021) can provide an integrative bioinformatic analysis environment that can capture a cross-domain functional representation of EVs based on multi-omics data. These temporal-spatial informative outcomes can help explain the origin, transfer, and delivery of diverse molecular cargo in the context of biomedicine. This in turn can support a rapid response in the clinical chain of prevention, diagnosis, prognosis, and treatment of human maladies such as major respiratory infectious diseases.

## Data Availability

The original contributions presented in the study are included in the article/supplementary material, further inquiries can be directed to the corresponding authors.
